# Challenges in Optimising the Successful Construction of Antibody Drug Conjugates in Cancer Therapy

**DOI:** 10.3390/antib7010011

**Published:** 2018-02-13

**Authors:** Thomas Mehrling, Daniel Soltis

**Affiliations:** 1 Mundipharma EDO GmbH, Basel CH-4020, Switzerland; 2 Consultant, Cleveland Heights, OH 441188, USA; dan.soltis@edoncology.com

**Keywords:** antibody-drug conjugate, smart chemotherapy, targeted treatment, solid tumours, haematological malignancies

## Abstract

Although considerable progress has been made in the field of cancer chemotherapy, there remains a significant unmet medical need, with a requirement to move away from traditional cytotoxics and explore novel, smarter chemotherapeutic approaches. One such example of the smart chemotherapy approach is antibody-drug conjugates (ADCs), which consist of an antibody that binds selectively to a cancer antigen linked to a cytotoxic agent. When developing an ADC, it may be necessary to produce a variety of constructs to fully assess the optimal configuration for the molecule. By testing ADCs prepared using a range of cytotoxic agents, linkers, or different antibodies, it is possible to fully assess the optimal approach for this treatment modality before advancing to the clinic. Since the development and approval of first-generation ADCs, significant improvements in development technology have occurred. Here, we consider the advances made within the field of ADCs, focusing on the development of EDO-B278 and EDO-B776, both of which have demonstrated efficacy in preclinical testing. Although some limitations remain in this field of development, the potential reduction in toxicity offered by ADCs justifies the investment in research to find workable solutions that could ultimately provide patients with superior outcomes.

## 1. Introduction

The field of cancer chemotherapy has advanced recently, with the development of highly potent small-molecule agents; however, non-specific toxicity, due to the actions of the agents on rapidly dividing cells, continues to be problematic, reducing the therapeutic window [1]. In addition, based on the results obtained from years of use in clinical practice of similar cancer chemotherapeutic agents, tumour cells may develop resistance to these agents, further reducing their utility [1]. There remains, therefore, a significant unmet medical need, with a requirement to move away from traditional cytotoxics and explore novel, smarter chemotherapeutic approaches.

**Figure 1:**
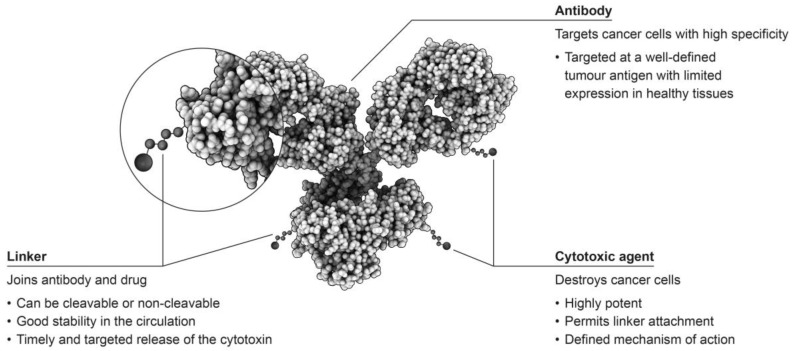
The structure of antibody-drug conjugates (ADCs) [1,2,3].

Antibody-drug conjugates (ADCs) consist of an antibody linked to a cytotoxic agent, which is sometimes referred to as the ‘payload’, and form some of the most sophisticated options for the treatment of tumours (Figure 1) [2,3]. This targeted approach has the potential to selectively attack only cells that are malignant, while leaving healthy cells unaffected, providing improved patient outcomes with fewer adverse events than observed with traditional chemotherapeutic approaches [4].

One of the earliest ADCs to receive marketing authorisation was brentuximab vedotin (Adcetris^®^, Seattle Genetics, Bothell, WA, USA), which was formed by the conjugation of the potent auristatin tubulin agent monomethyl auristatin E (MMAE) to an anti-CD30 antibody using a cleavable valine-citrulline dipeptide linker [5]. The objective response rate (ORR) in patients with relapsed or refractory Hodgkin lymphoma treated with brentuximab vedotin was shown to be 75% in a pivotal single-arm, Phase II study, with a median duration of response of 20.5 months [6]. These results are a significant improvement in outcome compared with previous treatment strategies. Moreover, in patients with systemic anaplastic large cell lymphoma, an ORR of 86% was observed [7].

The first ADC approved for the treatment of solid tumours was ado-trastuzumab emtansine (Kadcyla^®^, Roche, Basel, Switzerland), which was produced by conjugating the sulfhydryl group of the maytansinoid emtansine to the lysine amino groups of the anti-human epidermal growth factor receptor 2 (HER2) antibody [8,9]. Findings from the pivotal Phase III EMILIA trial in patients with HER2-positive metastatic breast cancer, who had progressed following treatment with a taxane plus trastuzumab, showed a superior ORR of 44% for those treated with ado-trastuzumab emtansine compared with lapatinib (31%) [10]. These patients also demonstrated progression-free survival (PFS) of 9.6 months, compared with 6.4 months in those who received lapatinib [10]. The initial success of this treatment approach sparked great interest in the technologies resulting in the initiation of a large number of development programs with different targets, but a limited number of linker/payload constructs.

A review published in 2016 reported that more than 50 putative ADCs are currently in clinical development, with approximately 20 candidates having been discontinued for a variety of reasons, including unforeseen or unacceptable toxicities [11].

## 2. Smart Chemotherapy: The Future for Cancer Treatment?

Smart chemotherapy aims to improve the targeting, efficacy and tolerability of new anti-cancer agents. ADCs are one example of a smart chemotherapy approach, maintaining the utility of cytotoxic agents with known efficacy and high potency, but combining this with targeted treatment of malignant cells while limiting toxicity on healthy cells in the body. Other examples of smart chemotherapy include multi-action therapies that bring together multiple modes of action within a single treatment [12], and improvements to drug pharmacokinetics through the development of prodrugs [13]. Such approaches move us closer to the goal of personalised medicine, where specific treatment approaches most appropriate to the individual’s tumour location and type are employed to optimise outcomes. One of the challenges facing smart chemotherapy in general, and ADCs in particular, is the identification of those patients most likely to respond favourably to any single treatment approach. Such identification is another facet of the smart chemotherapy approach, where it is hoped that the concomitant development of response predictors using techniques such as biopsies and molecular imaging will assist in tailoring treatment to the individual needs of the patient. Thus, the elaboration of strategies to identify relevant patient subgroups, including genetic profiling of the tumour and patient, and the use of immunohistochemistry (IHC) and fluorescence in situ hybridisation (FISH), alongside the development of novel treatments should be a priority. In combination, this approach should result in the production of molecules with the best possible risk/benefit profile for patients.

The intended mechanism of action for any ADC is that the chosen antibody binds selectively and efficiently to an antigen that is uniquely expressed on the tumour surface [3]. It is generally believed that the ADC is then internalised and degraded to release the cytotoxic component, which then induces cell death [3]; however, recent data suggest that there may be a potential applicability of non-internalising ADCs for cancer therapy [14]. It is this specific targeting of the tumour cells that provides the high therapeutic index associated with ADCs, and is a key feature determining the suitability of an antibody to be selected for conjugation with a cytotoxic agent. Therefore, antibodies known to demonstrate low affinity binding, or which are targeted to an antigen with low levels of expression, or which are not effectively internalised, are unsuitable for development as ADCs [11]. Thorough knowledge of antibody’s properties is essential, therefore, to ensure the creation of a successful research project to develop an ADC capable of moving from the bench to the bedside.

## 3. Optimising Composition of ADCs

As previously discussed, ADCs consist of three components, all of which should be defined and characterised. Optimal efficacy and tolerability depend on several steps in the process to successfully attack cancer cells in patients. Firstly, the antibody chosen for the construct should target a cell surface molecule that is either selectively expressed on cancer cells or overexpressed on cancer cells compared to healthy cells [15,16]. Although tumour-specific antigens would be ideal, this may not be possible for all tumour types, therefore, tumour-associated antigens with low expression on healthy cells may be preferred [1,16]. For example, prostate-specific membrane antigen (PSMA) is expressed in both normal and malignant tissue, however, in healthy prostate tissue PSMA is found only within the cytosol and so will not trigger ADC binding to healthy cells ensuring that normal tissues remain unaffected by the agent [17]. The level of antigen expression can also be a key component to ensuring optimal efficacy with ADCs. For example, for effective targeting of breast cancer cells, a high degree of overexpression of HER2 is required [18]. In addition, an antibody may not be a suitable candidate for construction of an ADC if it displays low affinity binding [11]. Once bound, the antibody needs to be efficiently internalised, and the payload, which should be tailored to the tumour type being targeted, released.

When developing a novel ADC, it may be necessary to produce a variety of constructs in order to fully assess the optimal configuration for the molecule. By testing ADCs prepared using a range of linkers and different antibodies, it is possible to more fully assess the optimal approach for this treatment modality before advancing to the clinic.

The chemistry of the chosen linker system is also a vital component in the rational design of an ADC. The linker must be sufficiently stable whilst in the circulation to allow the active moiety to remain attached to the antibody as it is distributed to the target tissues, and yet permit efficient release of the payload once internalisation into the malignant cell has occurred [14]. The stability of the linker can exert a considerable influence on the toxicities that might be associated with the active component of the ADC [11]. The most stable linkers will only release the chemotherapeutic component of the ADC in a target-specific manner; however, less stable linkers are prone to non-specific cleavage, resulting in a broader toxicity profile [11]. In addition, linkers can be classified as cleavable or non-cleavable, with cleavable linkers being those that are cleaved from the active component of the ADC by any of a variety of mechanisms including acidic degradation (hydrazones), protease cleavage by cathepsin B (dipeptide), and thiol-disulphide exchange reactions (disulphide), most of which occur in the endosomes of lysosomal compartments [19]. In contrast, non-cleavable linkers, such as maleimidocaproyl and thioether linkers, require complete lysosomal proteolytic degradation of the targeting antibody to occur, leaving the active component attached to the linker and a charged lysine or cysteine residue [19]. Importantly, an analysis of Phase I study data for several ADCs in development that was conducted by the FDA noted that ADCs utilising the same linker, but distinct target antigens, exhibited similar toxicity profiles, highlighting the importance of linker selection in agent development [3,16].

## 4. Assessing ADCs during Development

Specific ligand binding assays have been developed, which take into consideration the challenges presented by the dynamic and heterogeneous nature of many ADCs [20]. Such assays utilise multiple capture and detection reagents, specific for the framework of ADCs as an initial screening technique to assess binding affinity. Electrochemiluminescent techniques have been employed to further screen the ligand binding abilities of successful candidates [20]. In vitro cytotoxicity assays are also required, together with approaches to examine cellular accumulation, endosomal routing and activation/intracellular drug release [21].

It is also desirable to assess antibody internalisation, particularly given that it is desirable for the antigen target to be rapidly internalised and efficiently recycled to the cell surface to promote accumulation of the ADC in the cell [22]. In addition, unmodified monoclonal antibodies, and antibodies conjugated to form ADCs may internalise with varying efficiency, with more rapid internalisation of some ADCs having been observed [22]. Techniques such as flow cytometry and radiolabelled antibody studies are used widely to assess the internalisation of antibodies targeted at the cell surface into the cell itself [23]. Macro-confocal imaging may also be considered, as may direct and indirect cytotoxic assays of ADCs [23]. However, although these assays have been shown to be a reliable means for assessing internalisation, their complexities and relatively high costs often limit their application for screening of large antibody libraries [23].

Furthermore, when developing ADCs a number of early checks can be performed to facilitate optimisation of the resulting agent, including assessments for potential adverse events. One potential adverse event of ADC administration that must be assessed is immunogenicity, which can affect both the efficacy and safety of a biological drug [24,25]. At present, the body of literature on immunogenicity of ADCs is limited; however, bioanalytical techniques have been developed to allow assessment of immunogenicity following the tiered strategy often applied during the development of monoclonal antibody therapeutics, including enzyme-linked immunosorbent assays (ELISA) and electrochemiluminescence [24,26].

A full understanding of the pharmacokinetics of an ADC, and how this may impact efficacy and toxicity is a key component of ADC design and delivery [27]. Drug-to-antibody ratio (DAR) is the average number of drugs conjugated to an antibody in an ADC; an important attribute of this treatment modality which can affect efficacy, toxicity and pharmacokinetics. For example, low drug loading levels are likely to reduce the potency of the ADC. DAR is often dependent on the amino acid to which the drug is conjugated, for example, in non-specific conjugation there are approximately 40 lysines present within an IgG scaffold to which the drug can attach [28], with DARs between 6 and 14 reported, depending on the chosen linker [29]. In contrast, cysteine residues are far less prevalent with, for example, four exposed disulphides present on IgG1, providing a total of eight conjugation sites. Therefore, conjugation via this site would result in an ADC with a lower DAR [19], with DARs of 2, 4, 6 or 8 typically seen, depending on the linker chemistry [28]. However, although ADCs with higher DARs may appear more potent in vitro, the faster plasma clearance of highly conjugated antibodies can result in lower efficacy in vivo, with decreased drug loading associated with greater efficacy [30]. In addition, the hydrophobicity of the ADC can accelerate clearance, with the use of hydrophilic linkers shown to improve efficacy [31]. Therefore, the careful and informed selection of the correct conjugation chemistry, together with optimal drug loading will play a role in optimising the production of an ADC with the desired efficacy and toxicity profile.

## 5. Preclinical Efficacy of ADCs in Development

Many of the ADCs that are currently in development use either maytansine derivatives, such as DM1 or DM4, or auristatins (MMAE/MMAF) as the chemotherapeutic component [11]. Provided satisfactory in vitro data are obtained, initial testing of putative ADCs is performed using preclinical tumour xenograft models utilising cell lines derived from the cancer indication of interest. It has been suggested, however, that many existing preclinical murine models may not adequately predict the clinical activity and tolerability of ADCs [2]. This observation may in part occur due to differences in the in vivo stability of the linker in different model species, most notably, the decreased stability observed in rodents when compared with primates [3]. Nonetheless, other studies have demonstrated xenograft models to be clinically relevant with a clear correlation between the activity of the ADC observed in some animal models and that seen in Phase II clinical trials [32]. However, it should be noted that tumour models may not accurately represent all stages of tumour progression, and that the selection of a tumour cell line used for the xenograft, and the route of implantation should also be carefully considered [22]. Moreover, species cross-reactivity, particularly when the model demonstrates poor binding of the ADC to the antigen targets should be considered, with the use of knock-in mice if necessary to ensure similar binding affinity in the model to that expected in man [22].

## 6. Advances in ADC Development Technology

Since the development and approval of first-generation ADCs, significant improvements in development technology have occurred. Linker technologies have advanced, with experiences from first-generation ADCs underlining the importance of a suitably stable linker to optimising the efficacy of the ADC [11]. Extensive research is underway to develop novel linkers for use in newer ADCs that are systemically stable providing a good sustained half-life, while permitting effective release of the chemotherapeutic element of the molecule at the target site [3,16]. Moreover, linker design has been shown to impact on both the active and passive cell permeability of an ADC, with certain linkers being particular targets for multidrug transporters [3]. Therefore, strategic linker design can be employed to increase hydrophilicity, charge or stearic bulk and thus reduce binding of the payload within the ADC to multidrug transporters [3]. This approach may rescue efficacy, particularly in those tumours that exhibit multidrug resistance.

Furthermore, it has become apparent that there are many factors that can influence the toxicity of an ADC. As previously discussed, ADC toxicity can occur as a consequence of early release of the chemotherapeutic component of the molecule prior to reaching the target site, or effects caused by expression of the target antigen on healthy tissues [3]. Moreover, studies have revealed that toxicity may be associated with the heterogeneity of ADCs, particularly in terms of DAR [28,30,33,34]. This has resulted in the use of site-specific conjugation in an attempt to reduce the heterogeneity of ADCs and so reduce toxicity [28]. For example, a toxicity study conducted by the Redwood Bioscience team showed that a C-terminally tagged ADC was much better tolerated than the non-specifically conjugated ADC [35]. A multitude of site-specific conjugation strategies are currently under investigation, aided by advances in bioorthogonal chemistry and protein engineering [27,28]. The production of more homogeneous ADCs will ensure a more predictable efficacy and tolerability profile is achieved and will allow improved understanding of the factors governing linker degradation and drug release [28].

An additional factor that has been shown to influence toxicity of ADCs is hydrophobicity. In studies where hydrophobicity of an ADC has been held constant, the linker becomes more stable resulting in reduced toxicity, particularly in rodent tumour models [3]. The situation in humans, however, may be more complex, with clinical data suggesting an inverse relationship between the stability of the linker and the tolerability of the ADC [36]. For example, amongst disulphide linkers the most labile linker examined, in cantuzumab mertansine has been shown to have a maximum tolerated dose of 6.0–8.0 mg/kg every 3 weeks, while the intermediately labile linker in coltuximab ravtansine and the most stable linker in AMG 595 have maximum tolerated doses of 3.5–7.0 mg/kg and 3.6 mg/kg every 3 weeks, respectively [36].

Monoclonal antibodies were first produced in the 1980s as murine proteins, which had the potential to be immunogenic in humans and thus not suitable for long-term therapy [37]. Initial attempts to reduce this immunogenicity involved chimerisation to graft the murine antigen binding Fab regions of the antibody onto a human IgG backbone, with more recent technologies enabling the production of fully humanised antibodies [38]. Thus, advances in antibody technology have the potential to improve the tolerability of ADCs by reducing the risk of immunogenicity through the use of fully human antibodies, which are not then viewed by the body as ‘foreign’. However, evaluating the risk of immunogenicity of ADCs is far more complex than it is for monoclonal antibody therapeutics due to the potential for components of either the linker or payload to induce a humoral immune response [24]. Analysis of the immunogenicity of ado-trastuzumab emtansine used a risk-based, tiered approach that included screening and titration to detect anti-drug antibodies and attempted to identify which part of the ADC was responsible for the immune response [37]. This study observed a 5.3% incidence of immunogenicity to ado-trastuzumab emtansine, with antibodies to all components of the ADC identified [39]. Similarly, in Phase III studies of brentuximab vedotin, 6% of patients have been observed to develop persistent anti-drug antibodies [40]. At the present time a variety of potential ADC candidates are being evaluated in Phase II and Phase III clinical studies for the treatment of a range of tumour types (Table 1).

## 7. ADCs under Development by EDO

Mundipharma EDO GmbH (EDO) currently has two ADCs in late-stage preclinical development, EDO-B278 and EDO-B776. The antibodies within these ADCs were originally developed as radioimmunotherapeutics; but advances in ADC component technologies have made it preferable, in certain instances, to develop non-radioimmunotherapeutic ADCs with other classes of cytotoxic agents. EDO-B278 is designed to target tissue factor, which is over-expressed by many solid tumours, including prostate, colorectal, non-small cell lung (NSCLC), breast, melanoma, pancreatic, and gastric tumours [38,41,42,43,44,45,46,47,48]. There remains a high level of unmet medical need in several of these tumour types, with long-term prognoses remaining poor despite recent advances in treatment. Binding affinities, and anti-blood coagulation activities of a range of anti-tissue factor antibodies were initially evaluated, with four hybridomas producing murine monoclonal antibodies that bound with high affinity and have relatively fast association and slow dissociation rates (Table 2). The antibody portion of EDO-B278, which binds relatively quickly and with high affinity to malignant, but not normal tissues showed no inhibition of tissue factor-mediated blood coagulation in a two-stage partial thromboplastin time assay [38,41]. The antibody has also been shown to accumulate within NSCLC SW-900 cells and to inhibit growth of this tumour xenograft in a murine model [41].

EDO-B776 is an ADC targeting a fragment of cancer antigen 125 (CA125), which is being developed to treat ovarian cancer. Ovarian cancer treatment is a major unmet need with little progress in recent decades. CA125 is highly overexpressed in ovarian cancer and a part of CA125 is shed from the tumour resulting in fragments circulating in the blood [49,50,51]. This is the basis for CA125’s utility as a biomarker for diagnosis and progression of disease, and for monitoring the outcome following treatment. Shed CA125 can have a negative impact on the outcome of therapy because most antibodies that bind to CA125 may also bind to fragments of CA125 circulating in the blood of patients and limit clinical efficacy. For this reason, the antibody portion of EDO-B776 was selected for its ability to preferentially bind to the cell-associated portion of the CA125 protein that remains on the surface of the cancer cell after shedding. Preclinical testing has shown that EDO-B776 delayed tumour growth, and exhibits synergistic activity when given in combination with paclitaxel [52].

## 8. Conclusions

Although some limitations remain in this field of development, the potential reduction in toxicity offered by ADCs justifies the investment in research to find workable solutions that could ultimately provide patients with superior outcomes. Numerous challenges are encountered during the development of novel ADCs, from selecting an appropriate target, to ensuring efficient conjugation and choosing the optimal payload, many factors can influence the efficacy and tolerability of these new treatment modalities. With this in mind, it should be noted that despite a large number of ongoing clinical trials to assess a wide range of ADCs for a variety of oncologic indications, so far only four agents have successfully launched to market, one of which (Mylotarg^®^; Pfizer Oncology, New York, NY, USA) was approved in 2000, withdrawn in 2010, and reapproved in 2017. In the development of its ADCs, EDO has encountered some of the challenges outlined in this article. Taking into account the considerable number of factors that influence the successful construction of an effective ADC, EDO is developing several new approaches in an attempt to overcome them. It is hoped that it will soon be possible to initiate clinical trials to examine fully the clinical characteristics of these ADCs, and that the promise demonstrated in preclinical studies will translate into a clinical benefit.

## Figures and Tables

**Table 1:** Antibody-drug conjugates (ADCs) currently under evaluation in Phase II and Phase III clinical studies (source: clinicaltrials.gov).

ADC	Target	Indication	Study Sponsor
**Phase III**			
Depatuxizumab mafodotin (ABT-414)	Epidermal growth factor receptor (EGFR)	Gliobalstoma Gliosarcoma	Abbvie, North Chicago, IL, USA
Mirvetuximab soravtansine	Folate receptor alpha	Ovarian cancer Peritoneal carcinoma Fallopian tube cancer	Immunogen Inc., Waltham, MA, USA
Polatuzumab vedotin	CD79b	Diffuse large B-cell lymphoma (DLBCL) Follicular lymphoma	Genentech, South San Francisco, CA, USA/Roche, Switzerland
Rovalpituzumab tesirine	DLL3	Small cell lung cancer	Abbvie, North Chicago, IL, USA
Sacituzumab govitecan (IMMU-132)	TROP-2 receptor	Triple negative breast cancer	Immunomedics Inc., Morris Plains, NJ, USA
SYD985	Human epidermal growth factor receptor 2 (HER2)	Metastatic breast cancer	Synthon Biopharmaceuticals, The Netherlands
Vadastuximab talirine	CD33	Acute myeloid leukaemia	Seattle Genetics, Bothell, WA, USA
**Phase II**			
AGS-16C3F	Ectonucleotide pyrophosphatase /phosphodiesterase family member 3 (ENPP3)	Renal cell carcinoma	Agensys Inc., Santa Monica, CA, USA;Astellas Pharma Inc., Japan
Anetumab ravtansine	Mesothelin	Lung neoplasms Pancreatic cancer	National Cancer Institute, Rockville, MD, USA
BMS-986148	Mesothelin	Mesothelioma Non-small cell lung cancer Ovarian cancer Pancreatic cancer Gastric cancer	Bristol-Myers Squibb, New York, NY, USA
CDX-014	TIM-1	Renal cell carcinoma	Celldex Therapeutics, Hampton, NJ, USA
Coltuximab ravtansine (SAR3419)	CD19	DLBCL	Sanofi, France
Denintuzumab mafodotin (SGN-CD19A)	CD19	Lymphoma, B-cell Lymphoma, large B-cell, diffuse Lymphoma, follicular, Grade 3b Follicular lymphoma, Grade 3b	Seattle Genetics, Bothell, WA, USA
DS-8201a	HER2	Colorectal cancer Gastrointestinal neoplasms Breast cancer	Daiichi Sankyo Co. Ltd., Japan
Enfortumab vedotin (ASG-22CE)	Nectin-4	Carcinoma, transitional cell Urinary bladder neoplasms Urologic neoplasms Renal pelvis neoplasms Urothelial cancer Ureteral neoplasms Urethral neoplasms	Astellas Pharma Global Development Inc., Northbrook, IL, USA
Glembatumumab vedotin	Glycoprotein NMB	Melanoma Osteosarcoma Metastatic gpNMB over-expressing Triple Negative Breast Cancer	Celldex Therapeutics, Hampton, NJ, USA
hLL1-DOX	CD74	Multiple myeloma	National Cancer Institute, Rockville, MD, USA
HuMax-AXL-ADC	Axl	Ovarian Cancer Cervical Cancer Endometrial Cancer NSCLC Thyroid Cancer Melanoma	Genmab, Denmark
Labetuzumab govitecan	CEACAM5	Metastatic colorectal cancer	Immunomedics Inc., Morris Plains, NJ, USA
Lorvotuzumab mertansine	CD56	Pleuropulmonary blastoma Recurrent malignant peripheral nerve sheath tumour Recurrent neuroblastoma Recurrent rhabdomyosarcoma Recurrent synovial sarcoma Wilms tumour	Children’s Oncology Group, Monrovia, CA, USA
PSMA ADC	Prostate Specific Membrane Antigen	Prostate cancer	Progenics Pharmaceuticals Inc., Tarrytown, NY, USA
RC48-ADC	HER2	Metastatic breast cancer	RemeGen
Sacituzumab govitecan (IMMU 132)	Tumor-associated calcium signal transducer 2 (TROP-2) receptor	Epithelial cancers	Immunomedics Inc., Morris Plains, NJ, USA
SAR566658 (ACT14884)	CA6	Triple negative breast cancer	Sanofi, France
SGN15	Lewis-Y antigen	Ovarian neoplasms	Seattle Genetics Inc., Bothell, WA, USA
Tisotumab vedotin (HuMax-TF-ADC)	Tissue factor	Ovarian Cancer Cervical Cancer Endometrial Cancer Bladder Cancer Prostate Cancer (CRPC) Oesophageal Cancer Lung Cancer (NSCLC)	Genmab, Denmark
Vadastuximab Talirine (SGN-CD33A; 33A)	CD33	Myelodysplastic Syndrome	Seattle Genetics Inc., Bothell, WA, USA
W0101	insulin-like growth factor 1 (IGF-1) receptor	Advanced solid tumours	Pierre Fabre Medicament, France

**Table 2:** Isotypes, affinities, and anti-blood coagulation activities of anti-human tissue factor monoclonal antibodies [38] (reproduced with permission from Chen et al. *Hybridoma.* 2005; 24:78–85).

BIAcore Analysis					Coagulation Time
Anti-TF MAbs	Isotype	ka (1/Ms)	kd (1/s)	KD (M)	Mean ± SD (s)
No Ab	N/A	N/A	N/A	N/A	185.0 ± 8.7
TF158	N/A	N/A	N/A	N/A	>450 ^a^
TF278	IgG1, *λ*	2.9 × 10^5^	1.5 × 10^−4^	5.2 × 10^−10^	190.0 ± 17.3
TF392	IgG1, *λ*	2.1 × 10^5^	2.3 × 10^−4^	1.1 × 10^−9^	210.0 ± 0.0
TF260	IgG1, *λ*	2.0 × 10^5^	2.6 × 10^−4^	1.3 × 10^−9^	185.0 ± 8.7
TF009	IgG1, *κ*	2.0 × 10^5^	3.6 × 10^−4^	1.8 × 10^−9^	195.0 ± 15.0
TF277	IgG1, *κ*	4.4 × 10^5^	3.1 × 10^−3^	7.1 × 10^−9^	205.0 ± 8.7
TF124	IgG1, *κ*	6.0 × 10^5^	1.5 × 10^−2^	2.5 × 10^−8^	200.0 ± 8.7
TF080	IgG1, *κ*	2.9 × 10^5^	2.0 × 10^−2^	6.8 × 10^−8^	202.5 ± 10.6
TF126	IgG1, *κ*	1.7 × 10^6^	1.8 × 10^−1^	1.0 × 10^−7^	ND
TF297	IgG1, *κ*	2.9 × 10^4^	7.1 × 10^−3^	2.4 × 10^−7^	225 ^a^
TF261	IgG1, *λ*	3.4 × 10^5^	1.0 × 10^−1^	3.0 × 10^−7^	225 ^a^
TF451	IgG1, *κ*	4.0 × 10^5^	1.5 × 10^−1^	3.6 × 10^−7^	180 ^a^
TF405	IgG1, *κ*	8.3 × 10^4^	3.4 × 10^−2^	4.1 × 10^−7^	220.0 ± 34.6

^a^ Single reading; N/A, not applicable; ND, not done due to high dissociation rates in BIAcore analyses.
