# Correction: Mehrling, T.; et al. Challenges in Optimising the Successful Construction of Antibody Drug Conjugates in Cancer Therapy. *Antibodies* 2018, *7*, 11

**DOI:** 10.3390/antib7030032

**Published:** 2018-08-27

**Authors:** Thomas Mehrling, Daniel Soltis, the Antibodies Editorial Office

**Affiliations:** 1 Mundipharma EDO GmbH, Basel CH-4020, Switzerland; thomas.mehrling@edoncology.com; 2 Consultant, Cleveland Heights, OH 441188, USA; dan.soltis@edoncology.com; 3 MDPI, St. Alban-Anlage 66, 4052 Basel, Switzerland

The Conflict of Interest section of the published paper [1] has been updated as follows:

“Thomas Mehrling is an employee of Mundipharma EDO, which is currently developing the ADCs EDO-B278 and EDO-B776. Daniel Soltis is a consultant to Mundipharma EDO.”

The manuscript will be updated and the original will remain online on the article webpage.
